# Exploring the Inhibitory Potential of Sodium Alginate Against Digestive Enzymes Linked to Obesity and Type 2 Diabetes

**DOI:** 10.3390/molecules30051155

**Published:** 2025-03-04

**Authors:** Chantal D. Daub, Arryn L. Michaels, Blessing Mabate, Lithalethu Mkabayi, Adrienne L. Edkins, Brett I. Pletschke

**Affiliations:** 1 Enzyme Science Programme (ESP), Department of Biochemistry, Microbiology and Bioinformatics, Rhodes University, Makhanda 6139, South Africa; chantaldaub97@gmail.com (C.D.D.); inferno.michaels@gmail.com (A.L.M.); bmabate@gmail.com (B.M.); lithalethum@gmail.com (L.M.); 2 Biomedical Biotechnology Research Unit (BioBRU), Department of Biochemistry, Microbiology and Bioinformatics, Rhodes University, Makhanda 6139, South Africa; a.edkins@ru.ac.za

**Keywords:** digestive enzyme inhibition, obesity, sodium alginate, type 2 diabetes mellitus

## Abstract

Obesity and type 2 diabetes mellitus (T2DM) are major health concerns worldwide, often managed with treatments that have significant limitations and side effects. This study examines the potential of sodium alginates, extracted from *Ecklonia radiata* and *Sargassum elegans*, to inhibit digestive enzymes involved in managing these conditions. We chemically characterized the sodium alginates and confirmed their structural integrity using FTIR, NMR, and TGA. The focus was on evaluating their ability to inhibit key digestive enzymes relevant to T2DM (α-amylase, α-glucosidase, sucrase, maltase) and obesity (pancreatic lipase). Enzyme inhibition assays revealed that these sodium alginates moderately inhibit α-glucosidase, maltase, and lipase by up to 43%, while showing limited effects on sucrase and α-amylase. In addition, the sodium alginates did not affect glucose uptake in human colorectal cells (HCT116), indicating they do not impact cellular glucose absorption. In summary, while the observed enzyme inhibition was moderate, the targeted inhibition of α-glucosidase, maltase, and lipase suggests that sodium alginates could be beneficial for managing postprandial hyperglycemia and lipid absorption in the context of T2DM and obesity.

## 1. Introduction

Obesity and type 2 diabetes mellitus (T2DM) are closely linked metabolic disorders, each exacerbating the other in a complex cycle [1]. Obesity is a significant risk factor for the development of T2DM, with the accumulation of visceral fat contributing to insulin resistance, an underlying feature of both conditions [2]. This resistance elevates blood glucose levels, driving the onset and progression of diabetes [3]. In turn, T2DM can also precede obesity in individuals with inherent insulin resistance. In these cases, increased hepatic glucose production and elevated insulin levels contribute to the development of obesity, rather than the reverse [1]. Given these intricate connections, current therapeutic strategies for managing both obesity and T2DM often employ common approaches, including lifestyle interventions, pharmacotherapy, and, in more severe cases, surgical procedures [1]. While these treatments can be effective, they often come with challenges such as poor patient adherence, side effects, and the invasive nature of surgical options [4]. Although modifying lifestyle factors like diet and exercise is essential for managing these conditions, many individuals struggle to maintain these changes long-term [4]. As a result, pharmaceutical therapies are frequently used to complement lifestyle modifications. In terms of available pharmacological treatments, blocking the digestion and absorption of dietary lipids and carbohydrates through enzyme inhibition offers a valuable alternative to traditional approaches for managing obesity and diabetes [5]. However, commercially available anti-obesity agents like orlistat and anti-diabetic agents such as acarbose which inhibit relevant enzymes are not without limitations. While effective, these medications are often associated with adverse side effects, such as gastrointestinal discomfort, which can reduce patient adherence and undermine their overall effectiveness [6,7]. Consequently, there is a growing interest in exploring alternative therapeutic agents, such as natural enzyme inhibitors, which may provide safer, more sustainable options for managing these conditions.

Sodium alginate, a polysaccharide derived from brown seaweeds, is widely recognized for its gel-forming properties, making it valuable in the food and pharmaceutical industries [8]. In addition to its physical properties, sodium alginate also exhibits bioactive effects that are relevant for managing obesity and type 2 diabetes [6]. One key action is its ability to swell in the stomach due to its viscosity, contributing to appetite suppression and potentially reducing energy intake, as observed in overweight males [3]. Beyond appetite regulation, sodium alginate has been shown to inhibit pancreatic lipase [6], an enzyme responsible for breaking down dietary triglycerides into absorbable free fatty acids and monoglycerides [9]. This inhibition reduces fat absorption, potentially aiding in weight loss. Notably, Wilcox et al. [10] observed that the extent of this inhibition is influenced by the frequency of mannuronate and guluronate residues in the binary copolymer structure. Furthermore, sodium alginate has demonstrated inhibitory effects on carbohydrate-digesting enzymes, including α-amylase and α-glucosidase [11,12], which are responsible for breaking down complex carbohydrates into glucose. By delaying carbohydrate digestion, alginate may help mitigate postprandial glucose spikes [13].

This study evaluates the inhibitory potential of sodium alginates from *Ecklonia radiata* and *Sargassum elegans* against digestive enzymes linked to obesity and type 2 Diabetes Mellitus (T2DM). By examining their effects on pancreatic lipase, α-amylase, α-glucosidase, sucrase and maltase, the study aims to assess their potential as natural agents for slowing down the digestion of carbohydrates and fats, thereby reducing postprandial hyperglycemia and aiding in weight management.

## 2. Results and Discussion

### 2.1. Alginate Yield and Composition

To evaluate *E. radiata* and *S. elegans* as potential sources of bioactive sodium alginate, we first extracted and characterized the alginates from these seaweeds. The results, including yield and chemical composition, are summarized in Table 1. For comparative purposes, the composition of commercially purchased sodium alginate from Sigma-Aldrich (St. Louis, MO, USA) was determined alongside our seaweed-derived sodium alginate extracts. The extraction of sodium alginate from *E. radiata* and *S. elegans* yielded a significant amount of the polysaccharide, with average yields around 40% (*w*/*w*) of the dry seaweed biomass. This yield aligns with previous studies, which indicate that alginate accounts for up to 40–47% of a seaweed’s dry weight [14].

The chemical composition analysis revealed that the sodium alginates extracted from both seaweed species were similar to the commercial alginate from Sigma-Aldrich. As detailed in Table 1, the alginates primarily consist of uronic acids, specifically D-mannuronic acid and L-guluronic acid, which are the key components of alginate [15]. The total uronic acid content in our extracts, around 35%, is lower than the ~49% found in the commercial alginate, but is comparable to the 27% to 29% reported for alginates from other commercially exploited brown algae species such as Laminaria digitata and Macrocystis pyrifera [15]. Notably, the *E. radiata* sodium alginate also contained a small amount of fucose (0.84%), suggesting the potential co-extraction of fucoidan, a compound known for its bioactive properties [7]. The M/G ratios, reflecting the ratio of D-mannuronic to L-guluronic acid, were greater than 1 for all alginates, indicating a predominance of D-mannuronic acid. This predominance is consistent with previous reports on *E. radiata*-extracted sodium alginate and the commercial Sigma-Aldrich alginate [16,17]. These findings confirm the identity of the sodium alginate extracts, as their composition closely matches that of the commercial alginate.

Alginates naturally occur within complex matrices in the cell walls and intercellular spaces of brown seaweeds and are often co-extracted with other carbohydrates, polyphenols, and proteins [18,19]. Consequently, assessing the purity of alginate extracts is essential to determine their suitability for therapeutic or industrial applications. In this study, we compared the composition of our sodium alginate extracts from *E. radiata* and *S. elegans* with a standard commercial alginate from Sigma-Aldrich. The impurity profiles were found to be comparable across all samples (Table 1). The low levels of L-fucose (<0.62%) and the absence of detectable sulfate groups indicate minimal contamination by fucoidan, a sulfated polysaccharide commonly co-extracted with alginates [19]. This suggests the effective separation of fucoidan during the extraction and purification processes. Similarly, the negligible glucose content (<1%) points to the minimal presence of cellulose and/or laminarin, which are structurally similar carbohydrates often extracted along with alginates. The protein content in the extracts was low (<1.54%), indicating that protein contamination was effectively minimized. Likewise, phenolic compounds were detected at very low levels (<0.08%), further suggesting that the extraction process successfully removed non-alginate organic contaminants.

### 2.2. Molecular and Physicochemical Properties of Sodium Alginate Extracts

Following the chemical composition analysis, additional molecular properties of the sodium alginates were examined to assess their relevance for enzymatic studies. Molecular weights (Mw) of the sodium alginate extracts were determined via high-performance size-exclusion chromatography (HPSEC). As summarized in Table 2, the extracted alginates exhibited molecular weights ranging from 194.27 kDa to 304.27 kDa, compared to the significantly lower molecular weight of the commercial sodium alginate (78.49 kDa). These values align with reported literature ranges for sodium alginate (48–756 kDa) [15,20,21]. Despite their inherent polydispersity, the extracted alginates displayed a narrow size distribution (PDI = 1), suggesting effective mitigation of polysaccharide depolymerization during the extraction process [22].

The kinetic viscosity analysis of the sodium alginate extracts revealed that the *E. radiata* alginate is more viscous than the *S. elegans* and commercial alginates, which showed a similar viscosity of between 1.96 and 1.98 cSt (Table 2).

In addition, the ash content of the sodium alginate extracts was profiled using thermogravimetric analysis (TGA). The extracted alginates exhibited ash contents exceeding 30.92%, markedly higher than the 22.38% observed in the commercial sodium alginate (Table 2). These results indicate a greater presence of mineral salts in the alginate extracts [23]. The elevated ash content in the extracted alginates can be attributed to various factors, including the source seaweed species, environmental conditions, and extraction methods [24].However, studies with similarly high ash contents have frequently pointed to residual salts used during the precipitation process as the most likely source [23,24,25,26]. In this case, the use of sodium carbonate (Na_2_CO_3_) in alginate precipitation likely contributed to the elevated mineral residue levels.

### 2.3. Thermogravimetric Analysis (TGA) of Sodium Alginates

**Figure 1:**
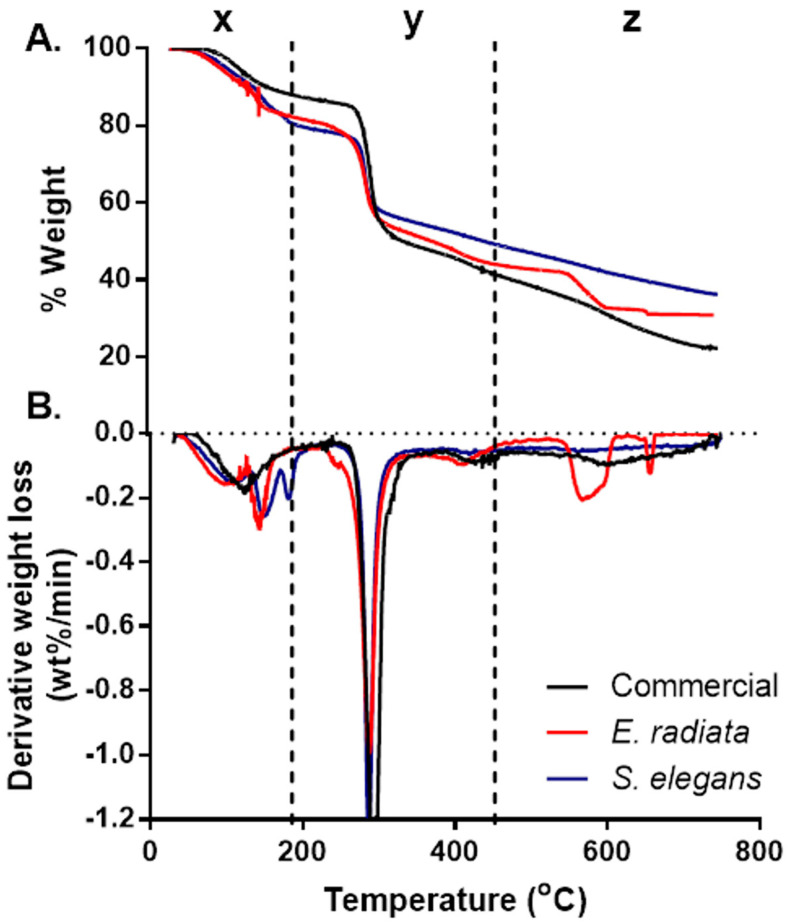
Thermogravimetric (TGA) (**A**) and derivative thermogravimetric (DTG) (**B**) curves of the sodium alginates. The thermal profiles highlight the distinct degradation stages and mass loss patterns, comparing *E. radiata*, *S. elegans*, and commercial sodium alginate samples.

Thermogravimetric analysis (TGA) was conducted to assess the ash content and thermal stability of the sodium alginate samples, with the resulting TGA and derivative thermogravimetric (DTG) curves presented in Figure 1 Three distinct degradation stages were identified, consistent with patterns reported for sodium alginates in previous studies [24,27].

The initial degradation stage, denoted as x, occurred between 30 °C and 180 °C, resulting in a mass loss of approximately 8–17%. This stage is attributed to the evaporation of physically adsorbed and hydrogen-bonded water molecules within the alginate matrix [27]. The second degradation stage (y), observed between 221 °C and 317 °C, represented the most significant mass loss (Figure 1). This phase corresponds to the thermal decomposition of the alginate polymer backbone. Among the samples, the commercial sodium alginate exhibited the highest weight loss (47%), followed by the *E. radiata* (41%) and *S. elegans* (36%) extracts. These differences may reflect variations in the molecular structure and purity of the alginate samples, particularly regarding their mannuronic to guluronic acid ratios [28]. The final degradation stage, denoted as z, was observed between 580 °C and 620 °C and was particularly pronounced in the *E. radiata* extract (Figure 1). This phase is associated with the decomposition of residual sodium carbonate (Na_2_CO_3_), a byproduct often formed during alginate processing [24], the thermal degradation profiles of the sodium alginate extracts closely mirrored those of the commercial standard, which is consistent with findings from previous studies [25], further supporting the similarity in thermal stability between the extracted and commercial alginates.

### 2.4. FT-IR Spectroscopy Analysis of Sodium Alginate

**Figure 2:**
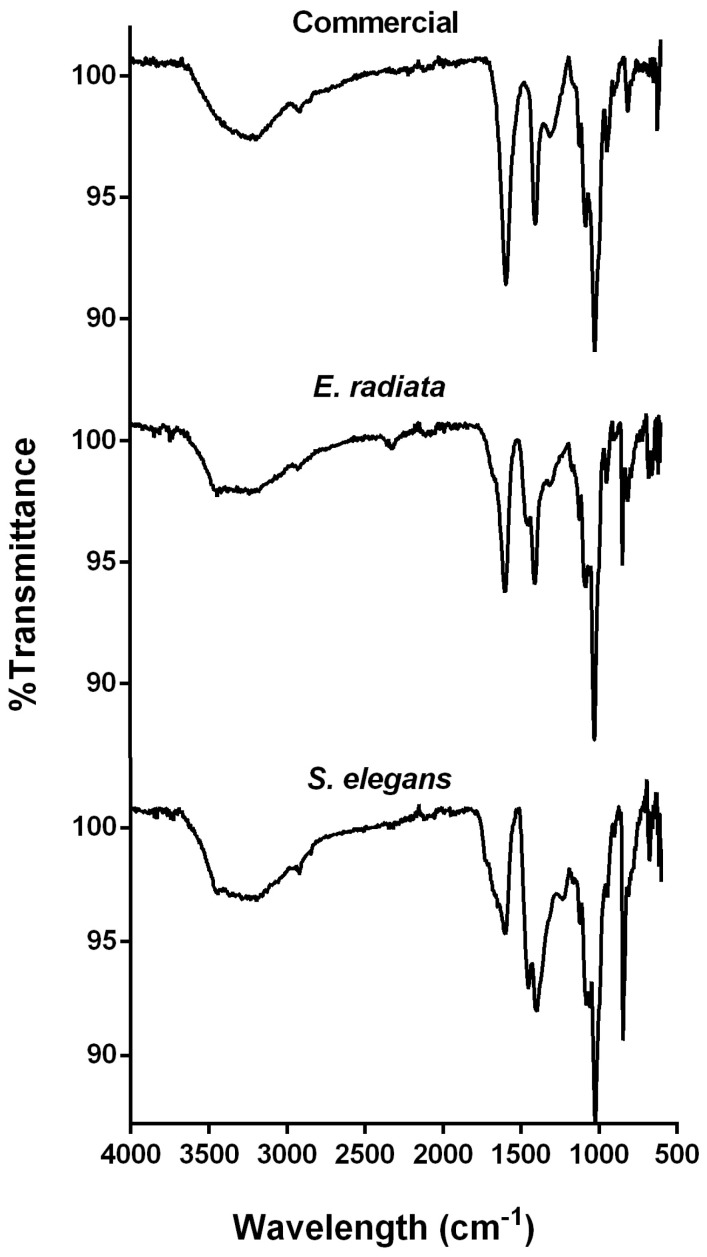
FTIR spectra of sodium alginates extracted from the brown seaweeds *E. radiata* and *S. elegans*, compared to the commercial sodium alginate standard.

FTIR analysis was employed for additional compositional and structural characterization of the alginate extracts. The FTIR spectra of the investigated extracts, compared to the commercial sodium alginate, exhibited characteristic absorption bands associated with sodium alginate (Figure 2). A broad, prominent band at 3400 cm⁻^1^, corresponding to O-H functional groups, and a smaller peak at 2900 cm⁻^1^, indicative of aliphatic C-H stretching, were observed [29]. The peaks at 1650 cm⁻^1^ and 1460 cm⁻^1^ were attributed to the asymmetric and symmetric stretching vibrations of carboxylate groups (O-C-O) in the alginate molecule [15,29]. Additionally, the bands at 1100 cm⁻^1^ and 1020 cm⁻^1^ corresponded to the C-O vibrations of pyranose rings, along with contributions from the deformation of C-C-H and C-O-H linkages in the mannuronic and guluronic acid units [15,30,31]. The band at 816 cm⁻^1^, characteristic of the flexion vibration of mannuronic acid residues, was also observed (Figure 2), confirming the presence of a significant mannuronic acid content [8,17]. Furthermore, the absence of a signal around 1200 cm⁻^1^, typically indicative of sulfate groups (S=O stretching), a hallmark of fucoidan and sulfated polysaccharides, confirmed the absence of such contaminants in the extracted alginates [32]. Overall, the FTIR spectra demonstrate structural similarities between the extracted alginates and the commercial standards.

### 2.5. NMR Analysis of Extracted Sodium Alginates

**Figure 3:**
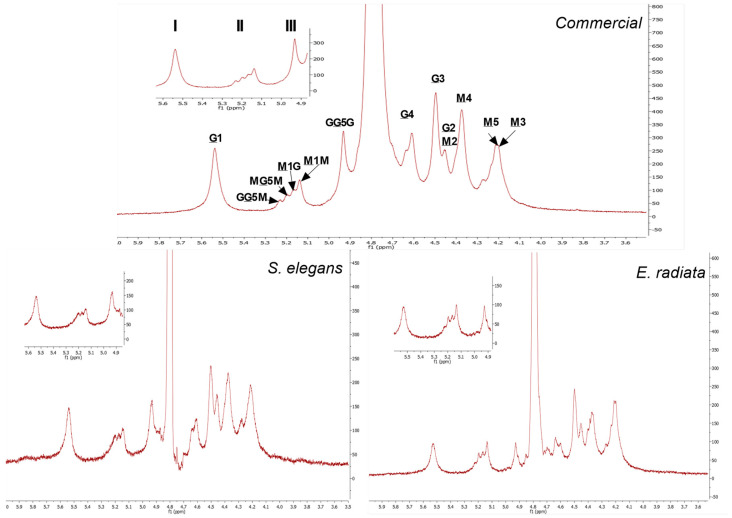
^1^H NMR spectra at 70 °C of commercial sodium alginate and alginate extracted from *E. radiata* and *S. elegans* harvested from the South African coastline. The underlined M and G denote proton signals corresponding to D-mannuronic acid (M) and L-guluronic acid (G) residues, respectively. Non-underlined letters represent neighboring residues along the copolymer chain. The numbers indicate the specific protons responsible for each signal in the spectra.

^1^H NMR was used to further elucidate the block structure profiles of the sodium alginates, see Figure 3. The ^1^H NMR spectra obtained showed clearly distinguishable signals for the mannuronic (M) and guluronic acid (G) units. The signal peaks representing the protons (H1–H5) located at the different carbon positions on the uronic acids (M and G) were assigned as asserted by the literature [32,33,34]. The mannuronic acid residue peaks denoted as M1, M2, M3, M4, M5 showed peak signals at 5.14 ppm, 4.45 ppm, 4.20 ppm, 4.27 ppm and 4.22 ppm, respectively. Furthermore, the spectrum showed the five typical L-guluronic acids residue peaks G1, G2, G3, G4, G5 at 5.54 ppm, 4.45 ppm, 4.50 ppm, 4.62 ppm and 4.93 ppm, respectively (Figure 3). The chemical shifts of the extracted alginates are similar to those of the commercial alginate.

In addition to the peaks representing the individual M and G components, the spectral resolution showed diad and triad frequencies in the sodium alginate polymer chains. The signals at 5.24 ppm, 5.20 ppm and 4.93 ppm represent the H5 proton of a central G residue in the G-centered triads (GGM, MGM) and homopolymeric G blocks, respectively. The M-diad signals M1G at 5.17 ppm and M1M at 5.14 ppm denote the M residues neighboring a G residue and another M residue [8]. The assessed alginates ^1^H NMR spectra exhibited no clear dominance of the peaks of the mannuronic acid monomers (M) over that of the guluronic acid monomers (G). However, the values in Table 3 showing the composition of sodium alginate determined by integration of signal areas, indicate that mannuronic acid (FM) was the main component in all species as well as the commercial alginate. The above results thus confirm the proximal composition detailed in Table 3.

### 2.6. Pancreatic Lipase Inhibition by Sodium Alginates

**Figure 4:**
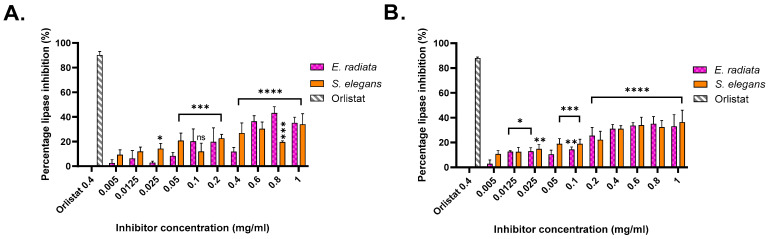
Pancreatic lipase inhibition by acid-extracted sodium alginates from brown seaweeds. (**A**) Inhibition activity against lipase using *p*-nitrophenyl butyrate as a substrate. (**B**) Inhibition activity against lipase using *p*-nitrophenyl octanoate as a substrate. Data represent the percentage inhibition of lipase activity by the extracted alginates compared to a control. Error bars indicate the standard deviation (*n* = 3). Statistical significance of inhibition level was assessed using two-way ANOVA, significance was defined as *p* value < 0.0001 (****), being more significant than *p* value > 0.0002 (***), 0.0021 (**) and 0.0332 (*), respectively.

The pancreatic lipase inhibitory activity of the extracted sodium alginates was evaluated using two substrates: *p*-nitrophenyl butyrate (*p*NPB), a short-chain triglyceride mimic, and *p*-nitrophenyl octanoate (*p*NPO), a medium-chain triglyceride mimic [35]. Absorbance measurements at 405 nm enabled the quantification of enzyme inhibition relative to an uninhibited reaction, with orlistat serving as the positive control. Orlistat exhibited approximately 90% inhibition on both substrates at a concentration of 0.4 mg/mL (Figure 4).

Among the alginate extracts, the highest inhibition on the *p*NPB substrate was achieved by sodium alginate from *E. radiata*, which exhibited an inhibition level of 43.18 ± 5.12% at a concentration of 0.8 mg/mL. On the *p*NPO substrate, the most significant inhibition was observed with sodium alginate from *S. elegans*, with an inhibition level of 36.40 ± 9.61% at 1 mg/mL. These results indicate moderate inhibitory potential for the alginates, consistent with previous studies, such as those by Wilcox et al. [10], which reported similar inhibition levels using alginates from *Laminaria hyperborea* on the synthetic substrate 1,2 Di-o-lauryl-rac-glycerol-3-(glutaric acid 6-methyl resorufin ester), DGGR.

The inhibitory activity of sodium alginates against *p*-nitrophenyl butyrate (*p*NPB) and *p*-nitrophenyl octanoate (*p*NPO) substrates highlighted a substrate-specific efficacy. Higher inhibition levels were observed for *p*NPB, indicating greater efficacy against shorter-chain triglycerides. The dose-dependent trend is evident for *E. radiata* and *S. elegans*, with increasing alginate concentrations resulting in greater inhibition, consistent with previous findings [6,10]. Although alginate inhibition was moderate compared to the anti-obesity drug orlistat, their natural origin and safety profile make them appealing for dietary interventions targeting obesity. Through statistical analysis, it was determined that the inhibition displayed by the extracts was significant; however, there was no statistical difference (*p* < 0.05) between the seaweed species in terms of their inhibition of pancreatic lipase (Figure 4).

### 2.7. Mode of Inhibition of Pancreatic Lipase by Sodium Alginates

The mode of inhibition was investigated to further understand the inhibitory mechanism of sodium alginates on pancreatic lipase activity. Enzyme kinetics, involving varying substrate and alginate extract concentrations, were used to determine whether the extracted alginates act as competitive, mixed, non-competitive or uncompetitive inhibitors.

**Figure 5:**
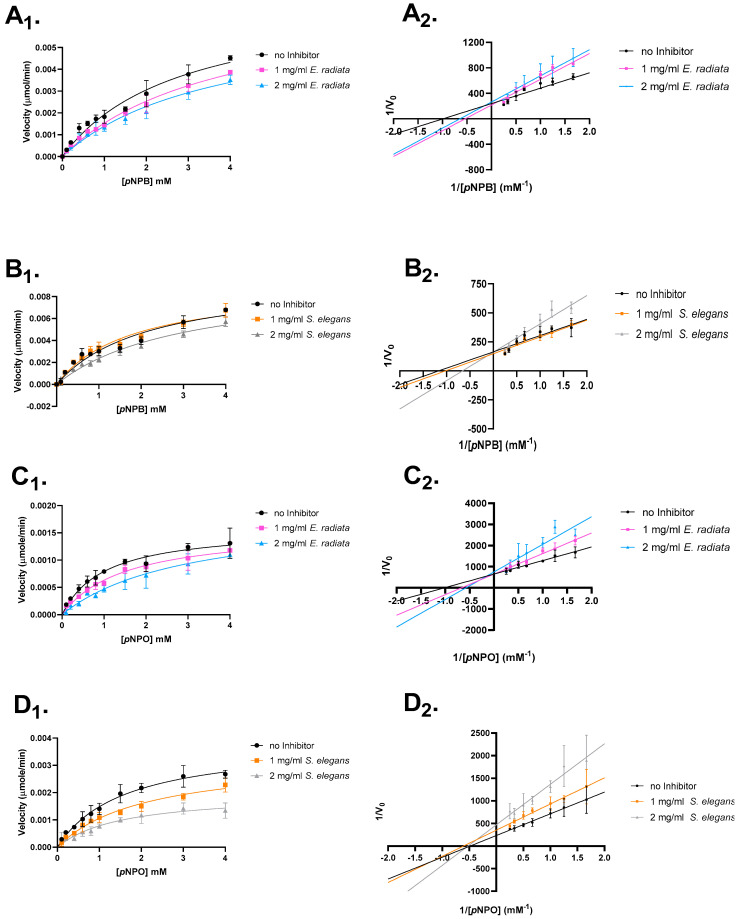
Mode of inhibition studies on pancreatic lipase. The effect of extracted sodium alginates on *p*-nitrophenyl butyrate and octanoate is shown. (**A**) *E. radiata* on *p*-nitrophenyl butyrate. (**B**) *S. elegans* on *p*-nitrophenyl butyrate. (**C**) *E. radiata* on *p*-nitrophenyl octanoate. (**D**) *S. elegans* on *p*-nitrophenyl octanoate. (X_1_) respective Michaelis–Menten plot. (X_2_) respective Lineweaver–Burk plot. Error bars indicate standard deviations (*n* = 3).

The type of inhibition was analyzed through Lineweaver–Burk plots and Michaelis–Menten kinetics (Figure 5). The results revealed that the extracted sodium alginates primarily displayed competitive inhibition, with a decrease in the apparent affinity (*K_m_*) for the substrate and no reduction in the maximum reaction velocity (*V_max_*). Additionally, some evidence for mixed inhibition was observed for *S. elegans* sodium alginate (Figure 5 and Table 4).

The observed differences in inhibition types suggest that the sodium alginates from *E. radiata* and *S. elegans* interact with pancreatic lipase in distinct ways. The competitive inhibition observed with *E. radiata* indicates that its alginate primarily competes with the substrate for binding at the enzyme’s active site, without affecting the enzyme’s overall structure. In contrast, the competitive and mixed inhibition observed with *S. elegans* suggests that its alginate interacts with the enzyme at or near the active site, but also potentially influences the enzyme’s conformation in a way that impacts both substrate binding and catalytic efficiency. These findings highlight the potential for sodium alginates to bind to different regions of the enzyme, offering a range of mechanisms for modulating lipase activity [36].

### 2.8. Inhibition of Type 2 Diabetes-Relevant Digestive Enzymes by Sodium Alginates

**Figure 6:**
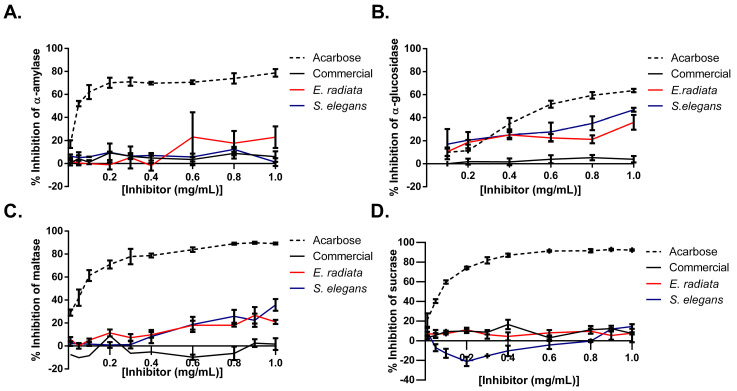
Inhibition of type 2 diabetes-relevant enzymes: (**A**) α-amylase, (**B**) α-glucosidase, (**C**) maltase, and (**D**) sucrase by sodium alginates and acarbose (positive control). Error bars indicate the standard deviation (*n* = 6).

Next, the inhibitory effects of sodium alginates on key digestive enzymes relevant to type 2 diabetes mellitus (T2DM) were evaluated by testing their activity against α-glucosidase, maltase, α-amylase, and sucrase. Acarbose, a standard anti-diabetic medication, was used as a positive control and demonstrated stronger inhibition across all enzymes, highlighting its broad inhibitory profile. In comparison, the sodium alginate extracts from *E. radiata* and *S. elegans* displayed selective inhibition, with the most pronounced effects on α-glucosidase (up to 40%) and maltase (~30%). In contrast, these alginates exhibited minimal to no inhibition of α-amylase and sucrase, indicating their specific targeting of the later stages of carbohydrate digestion (Figure 6). Notably, the commercial sodium alginate showed negligible inhibitory activity across all tested enzymes, further emphasizing the potential advantage of the alginate extracts for modulating postprandial glucose levels. The difference in activity between commercial sodium alginate and the alginate extracts from *E. radiata* and *S. elegans* could potentially be attributed to the commercial sodium alginate’s lower molecular weight (Table 2) and M/G ratio (Table 3), which may have reduced its ability to interact effectively with the enzymes. In addition, the unknown extraction conditions for the commercial sodium alginate might have involved harsher processing methods, which could have compromised its structural integrity and, as a result, limited its bioactivity.

While previous studies, such as Nakamura et al. [37] and Samudra et al. [11], have acknowledged the maltase- and α-glucosidase-inhibitory potential of sodium alginates, there are no other studies in the literature that have performed a similar screening of all four carbohydrate-digesting enzymes (Figure 6). Our findings confirm the inhibitory effects of sodium alginates on maltase and α-glucosidase and indicate a selective activity of alginates from *E. radiata* and *S. elegans*. The alginate extracts exhibited stronger inhibition against α-glucosidase and maltase, with negligible effects on α-amylase and sucrase. This selective inhibition may have resulted from specific interactions between the alginates and the active sites of α-glucosidase and maltase, while the negligible effects on α-amylase and sucrase could be due to structural differences in these enzymes, preventing effective binding of the alginates. This suggests that these alginates may specifically target the later stages of carbohydrate digestion. Such selective inhibition contrasts with broader-acting inhibitors, such as acarbose, which affect multiple enzymes involved in carbohydrate breakdown, including α-amylase. While acarbose is effective in managing postprandial glucose levels, its inhibition of α-amylase is also associated with side effects such as gastrointestinal discomfort, including bloating, flatulence, and diarrhea [7].

### 2.9. Fluorescent Quenching and Binding Interactions of α-Glucosidase with Sodium Alginate Extracts from E. radiata and S. elegans

**Figure 7:**
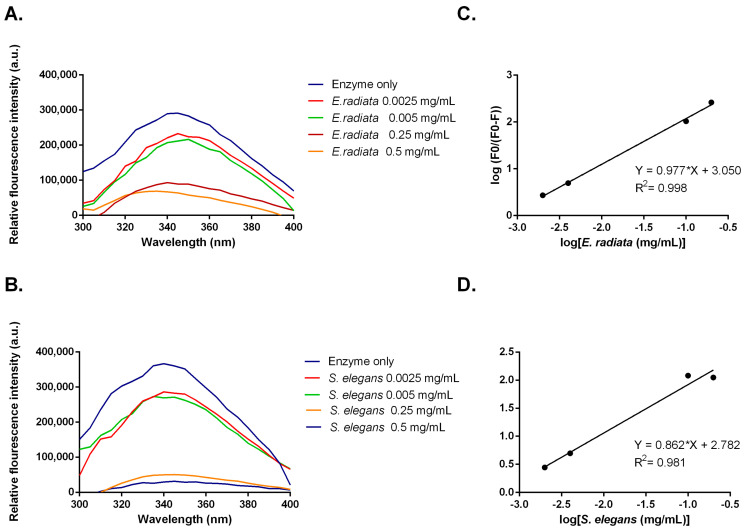
(**A**,**B**) Fluorescent emission spectra and (**C**,**D**) modified Stern–Volmer plots for fluorescent quenching of α-glucosidase by sodium alginate from *E. radiata* and *S. elegans*.

Inhibitors can alter the tertiary structure of α-glucosidases, which are detectable through changes in tryptophan fluorescence [38]. Measurements of α-glucosidase fluorescence, which contains exposed tryptophan residues, were taken in the presence of sodium alginates from *E. radiata* and *S. elegans*. The data showed a dose-dependent decrease in fluorescence intensity, indicating that sodium alginates bind to α-glucosidase, causing conformational changes (Figure 7). To further analyze this interaction, a modified Stern–Volmer plot was constructed (Figure 7). This plot, where the slope indicates the number of binding sites [39], revealed an *n* value of approximately 1. This suggests that the sodium alginates bind to a single site on α-glucosidase, indicating that the observed moderate inhibition might be due to specific binding at this site. Although viscosity has been shown to impact enzyme function in some cases [40], both the alginate extracts from *S. elegans* and the commercial sodium alginate in this study exhibited similar viscosities. However, only the *S. elegans* alginate demonstrated significant inhibition of enzyme activity. This suggests that viscosity alone was not responsible for the observed inhibition. Instead, the data in Figure 7 indicate that the inhibition is more likely due to direct, specific binding interactions between the alginates and the enzyme, rather than non-specific effects related to increased viscosity. While viscosity can influence enzyme activity by affecting diffusion rates [41], our results point to the primary cause of inhibition being the direct interaction of the alginates with the enzyme’s active site, as evidenced by the changes in enzyme activity.

### 2.10. Sodium Alginate Does Not Affect Glucose Uptake in HCT116 Colon Cancer Cells

Following the release of glucose by digestive enzymes, its uptake into cells is essential for reducing postprandial blood glucose levels. In type 2 diabetes mellitus (T2DM), this uptake process is impaired due to ineffective insulin utilization [42,43]. To evaluate the effect of sodium alginate on glucose uptake, we used the fluorescent glucose analogue 2-NBDG to assess glucose uptake in HCT116 human colorectal carcinoma cells. This cell line was chosen as it is relevant for studying oral sodium alginate administration, as dietary fibers like sodium alginate persist in the digestive tract for extended periods, especially in the colon, where they can remain for up to a day [44].

**Figure 8:**
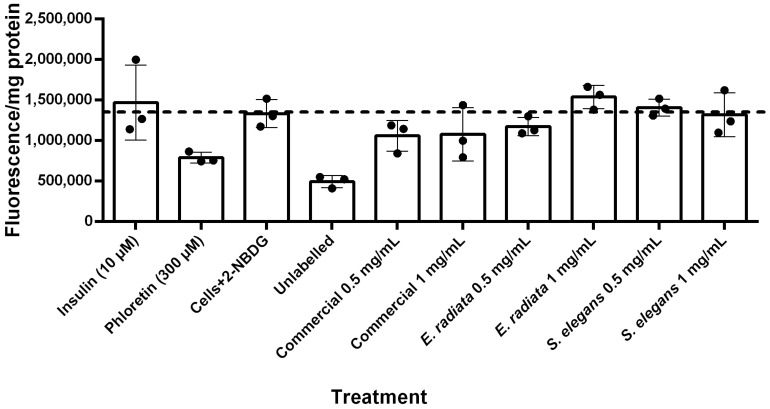
Glucose uptake in HCT116 cells treated with 2-NBDG under various conditions after 5 h incubation. Fluorescence intensity per mg protein is presented as mean ± SD (*n* = 3), with normalization for cell number based on protein content determined by the BCA (Bicinchoninic acid) protein assay.

Figure 8 shows the fluorescent quantification of 2-NBDG uptake into HCT116 cells. Unlabeled HCT116 cells (i.e., cells without 2-NBDG) exhibited minimal background fluorescence. The results indicated that insulin did not significantly increase glucose uptake in these cells. In contrast, phloretin, a known GLUT inhibitor [45], substantially reduced 2-NBDG fluorescence to levels comparable to those of unlabeled cells, demonstrating effective inhibition of glucose uptake. The sodium alginate extracts, however, did not significantly affect glucose uptake in HCT116 cells (Figure 8), suggesting that sodium alginate has no substantial impact on glucose uptake in this model. This lack of effect suggests that while these sodium alginates do not reduce glucose levels through increased uptake, they also do not promote excessive glucose absorption, thereby reducing the risk of hyperglycemia while still exerting their beneficial inhibitory effects on digestive enzymes.

## 3. Materials and Methods

### 3.1. Materials

Commercial sodium alginate (Cat. No. W201502), orlistat (CAS No. 96829-58-2), acarbose (Cat. No. A8980), Saccharomyces cerevisiae α-glucosidase (Cat. No. G5003), and porcine type II pancreatic lipase (CAS No. 9001-62-1) were purchased from Sigma-Aldrich. The other carbohydrate-digesting enzymes—porcine pancreatic α-amylase (Cat. No. E-PANAA-9G), maltase (Cat. No. E-MALTS), and sucrase (Cat. No. E-SUCR)—were obtained from Megazyme™ (Bray, WC, Ireland). Analytical kits used in this study were also sourced from Megazyme™. All other reagents were purchased from Sigma-Aldrich, Merck (Darmstadt, HE, Germany), and Megazyme™.

### 3.2. Seaweed Sampling Sites and Processing

The brown seaweeds *E. radiata* and *S. elegans* were harvested from separate locations in the Eastern Cape, South Africa: Middle Beach (−33°41′42.0102″ E; 26°40′1.7436″ S) and Kelly’s Beach (−33°36′37.1442″ S; 26°53′26.073″ E), respectively. The seaweed species identities were confirmed based on morphology by the taxonomic specialist Professor John Bolton (University of Cape Town, Cape Town, South Africa). The harvested seaweeds were rinsed, oven-dried at 50 °C, ground into a fine powder, and stored at room temperature until further use.

### 3.3. Sodium Alginate Extraction

Sodium alginate extraction was conducted according to Torres et al. [18] and Fertah et al. [8]. Dried pulverized seaweed samples were soaked in 2% (*v*/*v*) formaldehyde for 24 h to eliminate pigments, washed with distilled water and incubated in 0.2 M HCl for a further 24 h. After this, the samples were rewashed with distilled water, prior to being extracted under agitation at 40 °C for 5 h in 2% (*w*/*v*) sodium carbonate. Following the extraction, sodium alginate was precipitated with 80% (*v*/*v*) ethanol and collected by centrifugation (10,000× *g* for 10 min). The collected precipitate was purified by filtration through a nylon membrane (0.45 μm pore size, Whatman International Ltd., Maidstone, UK), washed with ethanol, methanol and acetone, and dried under ambient atmosphere, and the extraction yield (% *w*/*w*) was noted. Samples were collected for purity analysis following precipitation and after all wash steps.

$$Yield\;\left(\%\;w/w\right)=\frac{crude\;sodium\;alginate\;weight}{seaweed\;dryweight}$$

### 3.4. Alginate Characterization

#### 3.4.1. Chemical Composition Analysis

Protein content was measured by the Bradford’s method using bovine serum albumin (BSA) as a standard [46].Total phenolics were determined according to a modified Folin–Ciocalteu method using gallic acid as a standard [47]. The sulfate content was quantified using a modified gelatine–barium method [48]. The sugar and uronic acid contents were quantified after 2 M trifluoroacetic acid (TFA) hydrolysis of the sodium alginates at 100 °C for 10 h. Total reducing sugar content was determined according to the DNS (dinitrosalicylic acid) method [49]. The L-fucose, D-glucose and D-xylose contents were determined according to the microplate Megazyme™ assay kits (K-GLUC, K-FUCOSE, K-XYLOSE). Total uronic acid content was determined according to the microplate Megazyme™ assay kit (K-URONIC). The mannuronic and guluronic acid contents were quantified using a Shimadzu HPLC system (Shimadzu Corp, Kyoto, Japan) equipped with a diode array detector (DAD), where chromatographic separation was achieved on a Hypersil Gold™ Sax column (Phenomenex, Torrance, CA, USA) under ambient conditions. Elution was performed under isocratic conditions with a mobile phase of 50 mM phosphate buffer adjusted to pH 3.0 with ortho-phosphoric acid, at a flow rate of 0.8 mL/min over 10 min. The injected volume for the samples and standards was 10 μL, and the UV absorption of the effluent was monitored at 214 nm. Standard curves for mannuronic and guluronic acid were constructed to interpolate the amount of these respective uronic acids in the sodium alginate samples.

#### 3.4.2. Molecular Weight Determination

The molecular weights of the sodium alginates were determined using high-performance size-exclusion chromatography (HPSEC) with a refractive index detector (HPLC-RID). Chromatographic separation was achieved using a Shodex OHpak SB-806M HQ (8.0 mm I.D. × 300 mm) column (Showa Denko, Tokyo, Japan) with 0.1 M NaNO_3_ aq. as the mobile phase over 25 min at a flow rate of 0.5 mL/min. The column temperature was set at 30 °C. An injection volume of 20 μL was used for all samples as well as the pullulan standards (Shodex, Tokyo, Japan) used to construct the Log MW vs. retention time curve for molecular weight determination. The number molecular weight (Mn), molecular weight (Mw), and polydispersity index (PDI) were calculated as follows [50]:

$$Mn=\frac{\sum N_{i}M_{i}}{\sum N_{i}}$$

$$Mw=\frac{\sum N_{i}M_{i}^{2}}{\sum N_{i}M_{i}}$$

$$PDI=\frac{Mw}{Mn}$$

#### 3.4.3. Kinematic Viscosity Analysis

The kinematic viscosities of 1% (*w*/*w*) solutions of sodium alginates in dH_2_O were measured using a semi-micro glass capillary viscometer size 50 (Cannon-Manning (State College, PA, USA) at 25 °C. Samples were equilibrated to the required temperature for 30 min before taking measurements. To obtain kinematic viscosity in mm^2^/s (cSt), the efflux time in seconds was multiplied by the viscometer constant (K = 0.003992) using the following equation:

viscosity=viscometer constant (K)×time (s)

#### 3.4.4. Thermogravimetric Analysis

The thermogravimetric analysis (TGA) tests were performed on a Pyris Diamond model thermogravimetric analyser (*PerkinElmer*^®^, Waltham, MA, USA). Briefly, in an aluminum crucible, ~4 mg of sample powder was heated from 30 °C to 800 °C at a heating rate of 30 °C/min with a continuous supply of nitrogen (20 mL/min). A separate run using an empty aluminum crucible was conducted for baseline correction. The weight loss relative to the temperature increase was plotted as TGA curves, and the degradation rates at different temperatures were plotted as differential thermogravimetric (DTG) curves.

#### 3.4.5. Fourier Transform Infrared Spectrometer (FT-IR) Analysis

The sodium alginates’ FITR spectra were recorded on a PerkinElmer Spectrum 100 Fourier transform infrared spectroscopy (FTIR) system (PerkinElmer, Waltham, MA, USA) equipped with a ZnSe (zinc selenide) ATR crystal. Each sample was pressed uniformly against the sample spotting surface using a spring-loaded anvil. The FTIR spectra were recorded in the range of 4000 to 600 cm^−1^ with a resolution of 4 cm^−1^. Baseline and ATR corrections for penetration depth and frequency variations were carried out using a Spectrum^™^ One software (version 1.2.1) system (PerkinElmer^®^, Waltham, MA, USA).

#### 3.4.6. Nuclear Magnetic Resonance (NMR) Analysis

The sodium alginate samples (10 mg/mL) were dissolved in D_2_O, and insolubilized particles were removed by centrifugation at 13,000× *g* for 2 min prior to NMR spectrum acquisition. The ^1^H NMR spectra were recorded at 70 °C using a Bruker Avance III (Fällanden, ZH, Switzerland) 600 MHz NMR spectrometer (with a cryoprobe for variable-temperature measurements) equipped with TopSpin NMR software, version 3.6.5 (Bruker, Billerica, MA, USA). The block structure and M/G ratio was calculated from the area under the ^1^H NMR signal peaks denoted as (I–III) according to the calculation proposed by Grasdalen et al. [50], using the following equations:

FG=AI/(AII+AIII) 

FM=1−FG 

FGG=AIII/(AII+AIII)

FGM=FG−FGG

FMM=FM−FGM

M/G=FM/FG

### 3.5. Enzymatic Inhibition Studies

#### 3.5.1. Pancreatic Lipase Inhibition Assays

A modified *p*-nitrophenol assay by Gilham and Lehner [51] was used to determine pancreatic lipase activity on 4-nitrophenyl butyrate (*p*NPB) and 4-nitrophenyl octanoate (*p*NPO) substrates. A 50 mM sodium phosphate buffer with 0.5% Triton X-100 (pH 7) was used in these assays. A stock solution of 50 mM substrate was prepared in isopropanol and stored at 4 °C until required. A pancreatic lipase enzyme solution (1.2 mg/mL), and sodium alginates were prepared in the phosphate buffer. The concentrations of the sodium alginate stocks were 0.5 to 10 mg/mL. Positive control orlistat (4 mg/mL) stock was prepared in a 1:1 DMSO: phosphate-buffered solution.

Each reaction contained 25 µL of the respective sodium alginate stock or orlistat, followed by the addition of pancreatic lipase enzyme solution (0.12 mg/mL) and *p*NPB or *p*NPO substrate (1 mM) for a total reaction volume of 250 µL. The reactions were then incubated at 37 °C for 30 min in a low-light environment before the absorbance was measured at 405 nm at 2 min intervals over 30 min using a BioTek Epoch™ 2 Microplate spectrophotometer (BioTek, Winooski Vermont USA) with Gen 5™ software (version 3.05).

The uninhibited reaction, a substrate control (only substrate and buffer) and color controls (inhibitors and substrate, no enzyme) were included as reactions in these assays. The activities obtained were then processed and represented as % inhibition. The % inhibition was calculated according to the following formula:

$$\%\;inhibition=\left(1-\;\frac{absorbance\;of\;test-absorbance\;of\;colour\;control}{absorbance\;of\;uninhibited\;reaction-absorbance\;of\;substrate\;control\;}\right)\times 100$$

#### 3.5.2. Mode of Pancreatic Lipase Inhibition by Alginates

The type of inhibition the sodium alginates exert on pancreatic lipase was investigated on the 4-nitrophenyl butyrate (*p*NPB) and 4-nitrophenyl octanoate (*p*NPO) substrates. A pancreatic lipase enzyme solution (1.2 mg/mL) was prepared in 50 mM phosphate buffer with 0.5% Triton X-100 (pH 7.0) as required. Both substrates were prepared in isopropanol with multiple stock concentrations: 5; 10; 15; 30; 40; 50; 75; 100; 150; and 200 mM. Two stock concentrations of inhibitors were used, namely 1 mg/mL and 2 mg/mL for alginates.

To 96-well plates, 25 µL sodium alginate stocks, orlistat stocks or buffer were added in duplicate to 50 mM phosphate buffer with 0.5% Triton X-100 (pH 7.0), followed by the addition of pancreatic lipase enzyme solution (0.12 mg/mL) and lastly 5 µL *p*NPB or *p*NPO substrate solution for total reaction volumes of 250 µL. The plates were then incubated at 37 °C for 30 min in a low-light environment before the absorbance was measured at 405 nm using a BioTek Epoch™ 2 Microplate spectrophotometer (BioTek, Winooski, VT, USA) with Gen 5™ software (version 3.05). Controls included were a substrate control (only enzyme and buffer), color controls (inhibitors, no enzyme) and enzyme control (substate and buffer) for each substrate concentration of *p*NPB and *p*NPO.

#### 3.5.3. α-Amylase Assay

The inhibition of α-amylase (0.1 mg/mL) by the sodium alginates and positive control (acarbose) at various concentrations (0.1–1.0 mg/mL) was evaluated in a 400 µL reaction volume. The substrate utilized was 2% (*w*/*v*) potato starch in 50 mM sodium phosphate buffer (pH 7.0). The reaction mixtures were incubated at 37 °C for 30 min with gentle agitation at 70 rpm. The reaction was terminated by adding DNS reagent, and the reducing sugars released were quantified using the DNS method [49]. Inhibitor controls were included to correct for reducing sugars released due to the thermal degradation of the inhibitors during the DNS assay. A control reaction was prepared using the same procedure, with the inhibitor replaced by buffer. The inhibitory effects were expressed as a relative percentage of the control according to the following formula:

$$\%\;Enzyme\;inhibition=\frac{\left(reducing\;sugars\;released\;by\;control-reducing\;sugars\;released\;by\;test\;reaction\right)}{reducing\;sugars\;released\;by\;control}\;\times 100$$

#### 3.5.4. α-Glucosidase Assay

The inhibitory potential of the sodium alginates and positive control (acarbose) was assessed at various concentrations ranging from 0.1 to 1 mg/mL in 50 mM sodium phosphate buffer (pH 7.0). Their inhibition of α-glucosidase (0.625 μg/mL) activity was evaluated by monitoring the release of *p*-nitrophenol from the substrate *p*-nitrophenyl-α-D-glucopyranoside (pNPG, 2.5 mM) in a reaction volume of 500 µL. The reactions were incubated at 37 °C, and the release of *p*-nitrophenol was measured at 1 min intervals over 20 min at 405 nm using an Epoch™ 2 spectrophotometer (Bio-Tek, Winooski, VT, USA) with Gen 5™ software (version 3.05). Absorbance measurements were corrected for the contributions of the buffer, enzyme, substrate, and respective inhibitors. The amount of *p*-nitrophenol released was interpolated from a *p*-nitrophenol standard curve. A control reaction was conducted using the same procedure with the inhibitor replaced by buffer. The inhibitory effects were expressed as a relative percentage of the control according to the following formula:

$$\%\;Enzyme\;inhibition=\frac{\left(p-nitrophenol\;released\;by\;control-p-nitrophenol\;\;released\;by\;test\;reaction\right)}{p-nitrophenol\;released\;by\;control}\times 100$$

#### 3.5.5. Maltase Assay

The inhibitory activity of the sodium alginates and positive control (acarbose) on maltase was determined by incubating the potential inhibitors at final concentrations ranging from 0.1 to 1.0 mg/mL with maltase (0.175 U/mL) and isomaltotriose (15 mM) in a total reaction volume of 100 µL. The reaction was conducted at 37 °C for 30 min, after which the enzymatic activity was terminated by heating at 100 °C for 10 min. The glucose concentration released was measured using the glucose oxidase/peroxidase (GOPOD) kit, following the manufacturer’s instructions and a glucose standard curve. A control reaction was included to monitor enzyme activity without the inhibitor. The inhibitory effects were expressed as a relative percentage of the control according to the following formula:

$$Enzyme\;inhibition\;\%=\frac{\left(glucose\;released\;by\;control-glucose\;realeased\;by\;test\;reaction\right)}{glucose\;released\;by\;control}\;\times 100$$

#### 3.5.6. Sucrase Assay

The inhibitory effect of sodium alginate and the positive control (acarbose) on sucrase (0.001 U/mL) activity was evaluated at inhibitor concentrations ranging from 0.1 to 1 mg/mL, using a fixed concentration of sucrose (10 mM) in a total reaction volume of 100 µL. The reaction mixtures were incubated at 37 °C for 30 min, followed by heating at 100 °C for 10 min to terminate the reaction. The glucose concentration released was measured using the glucose oxidase/peroxidase (GOPOD) kit, following the manufacturer’s instructions and referencing a glucose standard curve. A control reaction was conducted to monitor enzyme activity without the inhibitor. The inhibitory effects were expressed as a relative percentage of the control according to the following formula:

$$Enzyme\;inhibition\;\%=\frac{\left(glucose\;released\;by\;control-glucose\;realeased\;by\;test\;reaction\right)}{glucose\;released\;by\;control}\;\times 100$$

### 3.6. Fluorescence Analysis of the α-Glucosidase–Inhibitor Interaction

The interaction between α-glucosidase and sodium alginate was analyzed using a modified steady-state fluorescence method, following [52]. Briefly, α-glucosidase (20 μg/mL) was incubated with sodium alginate at concentrations of 0.025 to 0.5 mg/mL in 0.05 M sodium phosphate buffer (pH 7.0) for 20 min at 37 °C. Fluorescence measurements were performed using a SpectraMax^®^ M3 Multi-Mode Microplate Reader (Separations, Molecular Devices, San Jose, CA, USA) at 25 °C with standard 96-well black microplates. Samples were excited at 280 nm, and emission spectra were recorded from 300 to 400 nm in 5 nm increments. Sodium alginate buffer controls were used to correct for background fluorescence. The Stern–Volmer equation, log ((F0 − F)/F) = logKa + nlog[Q] was applied to assess the number of binding sites, where F0 and F are the steady-state fluorescence intensities in the absence and presence of quencher, respectively, [Q] is the concentration of the quencher, and Ka and n are the binding constant and number of binding sites, respectively [39].

### 3.7. Determination of Alginates’ Effect on Cellular Glucose Uptake

#### 3.7.1. Cell Culture

The HCT116 human colon cancer cell line was purchased from the American Type Culture Collection (ATCC CCL-247). The cell line was cultured in Dulbeco’s Modified Eagle’s Medium (DMEM) with GlutaMAX™-I, 10% (*v*/*v*) fetal bovine serum (FBS), and 1% (*v*/*v*) penicillin, streptomycin and amphotericin (PSA), and was maintained at 37 °C with 9% CO_2_ in a humidified atmosphere.

#### 3.7.2. Glucose Uptake Assay

The glucose uptake assay utilized the glucose analogue 2-(N-(nitrobenz-2-oxa-1,3-diazol-4-yl)-amino)-2-deoxyglucose (2-NBDG) (Thermo Scientific, Waltham, MA, USA). Cells were seeded at a density of 5 × 10^5^ cells per well in a 96-well black-walled plate and allowed to adhere overnight. The following day, the cell culture medium was replaced with glucose-free DMEM containing 100 μM of 2-NBDG and treatments consisting of 10 μM insulin (positive control), 300 μM phloretin (negative control), or 500 and 1000 μg/mL sodium alginate. The cells were incubated for 5 h at 37 °C, then washed and resuspended in ice-cold phosphate-buffered saline (PBS). Fluorescence was measured in the FITC range (excitation: 465 nm; emission: 540 nm) using a SpectraMax^®^ M3 Multi-Mode Microplate Reader (Separations, Molecular Devices, San Jose, CA, USA).

Following fluorescence measurement, cells were lysed with 5× Triton X-100 for 20 min at 37 °C. The protein content of cell lysates (25 μL) was determined by adding 230 μL of BCA protein assay reagent (Thermo Scientific™, Pierce) in clear 96-well microtiter plates. Colorimetric readouts were obtained after 30 min of incubation at 37 °C using a PowerWaveX™ spectrophotometer (KC Junior software^®^, version 11) plate reader at 562 nm. Protein concentrations were derived from a BCA calibration curve constructed with varying concentrations of bovine serum albumin (BSA). Glucose uptake was reported as fluorescence (AU)/protein (mg) to account for variations in cell seeding between wells.

### 3.8. Statistical Analysis

The *t*-tests were performed using GraphPad Prism software version 10 (GraphPad Inc., Boston, MA, USA). Significant differences in glucose uptake and enzyme activity between the absence and presence of inhibitors were assessed using *t*-tests. The *t*-tests were conducted using the data analysis tools in GraphPad Prism software version 10 (GraphPad Inc.). Two-way ANOVA was used for the analysis of enzyme activity in the presence of inhibitors. Differences were considered statistically significant at *p* < 0.05.

## 4. Conclusions

In conclusion, this study demonstrates the potential of sodium alginates from *E. radiata* and *S. elegans* as selective inhibitors of digestive enzymes relevant to type 2 Diabetes Mellitus (T2DM) and obesity. The moderate inhibition of pancreatic lipase observed suggests a potential role for sodium alginates in obesity management by reducing the breakdown and absorption of dietary triglycerides, potentially limiting lipid accumulation and supporting weight management. Additionally, the alginates demonstrated effective inhibition of α-glucosidase and maltase, key enzymes involved in the final stages of carbohydrate digestion, suggesting their capacity to modulate postprandial glucose spikes. While the observed inhibition was less potent than orlistat and acarbose, the dose-dependent effects point to the potential of alginates as a natural, adjunctive approach to managing T2DM. Notably, the inhibition was shown to result from direct interactions between the alginates and the enzymes, highlighting the specificity of their action. Moreover, by inhibiting multiple key enzymes, alginates may have a cumulative impact that contributes to lowering hyperglycemia. With their selective inhibition and the potential for fewer side effects than current pharmaceuticals, sodium alginates represent a promising natural approach for supporting weight management and glycemic control.

## Figures and Tables

**Table 1:** Yield and chemical profiles of the sodium alginate extracts obtained from the brown seaweeds investigated and commercial sodium alginate. The data points represent mean values ± SD (*n* = 3).

Sodium Alginate Source	Commercial	*E. radiata*	*S. elegans*
Yield *	n.a.	37.71 ± 0.71	46.00 ± 6.89
Total protein ^a^	1.37 ± 0.51	0.88 ± 0.14	1.42 ± 0.54
Total phenolics ^a^	0.04 ± 0.00	0.088 ± 0.02	0.026 ± 0.01
Total reducing sugar ^a^	3.75 ± 0.01	4.78 ± 0.02	2.86 ± 0.02
L-fucose ^a^	ND	0.84 ± 0.29	ND
D-glucose ^a^	0.29 ± 0.12	0.72 ± 0.11	0.43 ± 0.00
Sulfate content ^a^	ND	ND	ND
Total uronic acid ^a^	49.28 ± 0.37	35.99 ± 5.96	35.85 ± 2.81
D-mannuronic acid ^b^	53.61 ± 4.36	54.24 ± 5.03	70.50 ± 4.34
L-guluronic acid ^b^	19.25 ± 2.51	16.48 ± 1.79	20.84 ± 1.27
M/G ratio	2.78	3.28	3.39

* Sodium alginate yield is given as percentage weight of seaweed dry weight; ^a^ all values are given as percentage composition of sodium alginate per weight; ^b^ mannuronic acid and guluronic acid content is represented as a percentage of total uronic acid content.

**Table 2:** Molecular characteristics and physicochemical properties of the sodium alginate extracts and commercial alginate. The data points represent mean values ± SD (*n* = 3).

Sodium Alginate Source	Mw * (kDa)	Mn * (kDa)	PDI * (M_w_/M_n_)	Kinematic Viscosity (cSt/s)	Ash Content (%)
Commercial	78.49	78.27	1.00	1.98 ± 0.02	22.38
*E. radiata*	304.24	295.70	1.03	2.70 ± 0.04	30.92
*S. elegans*	194.27	191.94	1.01	1.96 ± 0.05	36.29

* The abbreviations Mw, Mn, and PDI denote the molecular weight, number molecular weight, and polydispersity index, respectively.

**Table 3:** Compositional analysis of sodium alginates based on NMR spectroscopy.

Source	FG	FM	FMM	FGG	FGM = MG	M/G
Commercial	0.43	0.57	0.50	0.50	0.08	1.33
*E. radiata*	0.32	0.68	0.28	0.72	0.04	2.12
*S. elegans*	0.37	0.63	0.44	0.56	0.06	1.67

**Table 4:** Mode of inhibition of pancreatic lipase on *p*-nitrophenyl butyrate and *p*-nitrophenyl octanoate.

Substrate	Inhibitor	Inhibitor Concentration (mg/mL)	*K_m_* * (mM)	*V_max_* * (µmole/min)	Type of Inhibition
*p*-nitrophenyl butyrate	uninhibited	-	2.82	0.0074	-
	*E. radiata*	1	3.83	0.0074	Competitive
		2	3.71	0.0066	Competitive
	uninhibited	-	2.19	0.0098	-
	*S. elegans*	1	1.79	0.0091	No effect
		2	3.01	0.0095	Competitive
*p*-nitrophenyl octanoate	uninhibited	-	1.02	0.0016	-
	*E. radiata*	1	1.50	0.0016	Competitive
		2	2.96	0.0019	Competitive
	uninhibited	-	1.61	0.0039	-
	*S. elegans*	1	2.10	0.0033	Mixed
		2	1.54	0.0020	Mixed

* *K_m_* and *V_max_* indicate the Michaelis constant and maximum reaction velocity, respectively.

## Data Availability

The data presented in this study are available on request from the corresponding author.
